# Gender, Race and Parenthood Impact Academic Productivity During the COVID-19 Pandemic: From Survey to Action

**DOI:** 10.3389/fpsyg.2021.663252

**Published:** 2021-05-12

**Authors:** Fernanda Staniscuaski, Livia Kmetzsch, Rossana C. Soletti, Fernanda Reichert, Eugenia Zandonà, Zelia M. C. Ludwig, Eliade F. Lima, Adriana Neumann, Ida V. D. Schwartz, Pamela B. Mello-Carpes, Alessandra S. K. Tamajusuku, Fernanda P. Werneck, Felipe K. Ricachenevsky, Camila Infanger, Adriana Seixas, Charley C. Staats, Leticia de Oliveira

**Affiliations:** ^1^Department of Molecular Biology and Biotechnology, Biosciences Institute, Federal University of Rio Grande do Sul, Porto Alegre, Brazil; ^2^Graduate Program in Cell and Molecular Biology, Biotechnology Center, Federal University of Rio Grande do Sul, Porto Alegre, Brazil; ^3^Interdisciplinary Department, Federal University of Rio Grande do Sul, Tramandaí, Brazil; ^4^Management School, Federal University of Rio Grande do Sul, Porto Alegre, Brazil; ^5^Department of Ecology, Rio de Janeiro State University, Rio de Janeiro, Brazil; ^6^Department of Physics, Federal University of Juiz de Fora, Juiz de Fora, Brazil; ^7^Federal University of Pampa, Uruguaiana, Brazil; ^8^Institute of Mathematics and Statistics, Federal University of Rio Grande do Sul, Porto Alegre, Brazil; ^9^Department of Genetic, Institute of Biosciences, Federal University of Rio Grande do Sul, Porto Alegre, Brazil; ^10^Medical Genetics Service, Hospital de Clínicas de Porto Alegre, Porto Alegre, Brazil; ^11^Biodiversity Coordination, National Institute of Amazonian Research, Manaus, Brazil; ^12^Department of Botany, Institute of Biosciences, Federal University of Rio Grande do Sul, Porto Alegre, Brazil; ^13^Graduate Program in Management, Escola Superior de Propaganda e Marketing, São Paulo, Brazil; ^14^Department of Pharmacoscience, Federal University of Health Sciences of Porto Alegre, Porto Alegre, Brazil; ^15^Biomedical Institute, Fluminense Federal University, Niterói, Brazil

**Keywords:** motherhood and academia, women career, gender gap, racial bias, gender equity

## Abstract

The coronavirus disease 2019 (COVID-19) pandemic is altering dynamics in academia, and people juggling remote work and domestic demands – including childcare – have felt impacts on their productivity. Female authors have faced a decrease in paper submission rates since the beginning of the pandemic period. The reasons for this decline in women’s productivity need to be further investigated. Here, we analyzed the influence of gender, parenthood and race on academic productivity during the pandemic period based on a survey answered by 3,345 Brazilian academics from various knowledge areas and research institutions. Productivity was assessed by the ability to submit papers as planned and to meet deadlines during the initial period of social isolation in Brazil. The findings revealed that male academics – especially those without children – are the least affected group, whereas Black women and mothers are the most impacted groups. These impacts are likely a consequence of the well-known unequal division of domestic labor between men and women, which has been exacerbated during the pandemic. Additionally, our results highlight that racism strongly persists in academia, especially against Black women. The pandemic will have long-term effects on the career progression of the most affected groups. The results presented here are crucial for the development of actions and policies that aim to avoid further deepening the gender gap in academia.

## Introduction

As COVID-19 spreads around the globe, countries are facing different degrees of lockdown and social distancing (World Health Organization, 2020). In most affected countries, schools and universities have shifted from in-person learning to online classes and remote activities/work. The pandemic is also altering the work dynamics of many academics and scientists, especially parents of young children (Myers et al., 2020; Staniscuaski et al., 2020), who face the additional challenge of balancing remote work and domestic labor, which includes full-time childcare responsibilities. Since the pandemic outbreak, editors from a variety of respected scientific journals have warned the scientific community of the decreasing number of manuscript submissions authored by women despite the overall increase in total submissions driven by male authors (Viglione, 2020). The effect is even more striking for publications with women as first authors (Vincent-Lamarre et al., 2020). The aim of this study was to investigate whether gender, race and parenthood are associated with academic productivity during the COVID-19 pandemic.

The gender gap in science and academic careers is not new, and it has been previously exposed in many ways, such as in relation to career transitions (Lerchenmueller and Sorenson, 2018; Cardel et al., 2020), patent registration (Frietsch et al., 2009; Whittington, 2011; Hunt et al., 2013) and publications (Brooks et al., 2014). Additionally, high-status awards and positions are less likely to be given to women in science (Lunnemann et al., 2019), and a funding and salary gap is observed in several countries (Shen, 2013; Valentova et al., 2017; James et al., 2019), showing that gender equity in science is far from being achieved. Despite good intentions, the patterns and attitudes within academic settings work systematically against women (MIT Committee on Women Faculty in the School of Science, 1999). Often, merit-based systems of evaluation and career advancement have led to gender inequalities in academia (Krefting, 2003; van den Brink and Benschop, 2012). The top positions of institutional hierarchies are dominated by men, the gatekeepers who evaluate performance, which helps to maintain the male perspective (Acker, 2006; Treviño et al., 2018).

There are several factors that contribute to the underrepresentation of women in higher positions and leadership in science, from gender stereotypes to conscious prejudice to unconscious bias (Reuben et al., 2014; Gaston, 2015; Carli et al., 2016). However, one major factor influencing women’s career path in science is still an understudied topic: motherhood. Mothers continue to struggle for a place in academic and scientific landscapes (Isgro and Castañeda, 2015), and myths and misunderstandings on this subject misdirect efforts and resources intended to solve the problem (Verniers and Vala, 2018). Williams and Ceci (2012), studying the impact of motherhood on women’s careers, concluded that the effect of children on women’s academic careers is so remarkable that it eclipses other factors contributing to women’s underrepresentation in science. According to Whittington (2011), in academia, mothers are less likely to register patents than men and childless women, and Kyvik (1990) found that women with children younger than 10 years of age are considerably less productive than their male counterparts. Sustaining a career while being a mother is particularly challenging in highly masculinized areas, such as in STEM (Herman and Lewis, 2012). For instance, it has been shown that new parents (male and female) are significantly less likely than their childless peers to remain in STEM full time after their first child is born or adopted, with 23% of new fathers and 43% of new mothers leaving full-time STEM employment for other types of work or leaving the labor workforce entirely (Cech and Blair-Loy, 2019). The motherhood penalty in academia is a worldwide issue, but the acknowledgment of the problem by the academic community is very recent, and the development of effective actions and policies toward solving it is rather scarce. Gender-neutral policies that attempt to level the playing field by adjusting measures of productivity to account for early child rearing have been adopted in some institutions. However, such policies have unintended consequences that can actually hurt women (Antecol et al., 2018).

Remote work, when analyzed from the perspective of gender roles, has been viewed as a way to perpetuate gender inequality, as women usually carry the burden of both paid work and domestic responsibilities (Sullivan and Lewis, 2001). This phenomenon has been aggravated during the pandemic, as noted by Power (2020). The results obtained by Lyttelton et al. (2020) suggest that the unprecedented increase in telecommuting in response to COVID-19 has the potential to exacerbate gender inequalities in the formal labor market and the domestic division of labor, particularly when daycares, childcare facilities, and schools are facing extended closures. The gap in productivity between academics with and without children is growing, since support networks (i.e., schools and grandparents) were unavailable during the pandemic and childcare, including children’s learning, is most likely to be entirely parents’ responsibility. Garbe et al. (2020) demonstrated that the majority of parents devoted more than 1 h per day to supporting their child’s learning while schools were closed. Childcare is a task predominantly performed by women, including academics (Britton, 2014; Jolly et al., 2014; Sallee et al., 2016). For instance, a recent study found that mothers with young children have reduced their work hours four to five times more than fathers who worked with telecommuting during the pandemic (Collins et al., 2020). The same scenario was observed in academia in a study with American and European scientists, which showed that female scientists and scientists with young children were disproportionately affected in their time devoted to research (Myers et al., 2020).

Racial issues intersect with gender and parenthood and influence women’s representation in academia, where women of color face a double bias and multiple challenges in a racially stratified environment characterized by dysfunctional racial and gender hierarchies of predominantly white institutions (Gutiérrez y Muhs et al., 2012; Langin, 2019). Black female academics represent a very small portion of the overall faculty population, comprising only 2% of practicing scientists and engineers (National Science Foundation, 2015) and of full-time professors in research institutions (McFarland et al., 2019) in the US, for instance. In Brazil, Black women account for only 3% of PhD supervisors (da Silva, 2010; Morcelle et al., 2019). There are many reasons for this underrepresentation of Black women in science, including systemic racism, lack of representation and race-based stereotypes (McGee and Bentley, 2017). This is a major issue because diversity is a keystone for building high-quality and innovative science (Nielsen et al., 2017; Hofstra et al., 2020).

All of the evidence presented here reveals the urgency of shedding light on the full picture of the pandemic’s impact on the careers of female academics. It is expected that the gender gap in productivity will increase after the pandemic, but it is not clear whether mothers will be more impacted or whether underrepresented groups in science, especially Black women, will suffer a greater impact from pandemic-related circumstances. Additionally, the identification of the impacts in scientific communities in developing countries should be a top priority behind the design of mitigation policies aimed at building more inclusive research capacities.

To contribute to this urgent discussion, we report herein the impact of COVID-19-related social isolation on the academic productivity of scientists in Brazil, focusing on the influences of gender, parenthood, and race. We collected data via an online survey broadly disseminated across Brazilian regions and research institutions over a month-long period of social isolation. The survey was completed by 3,345 scientists. For the purpose of this study, academic productivity is regarded as the ability to submit papers within a schedule and to meet overall deadlines in the pandemic period. The design of the survey aimed to provide a comprehensive assessment of the various elements of academic productivity relevant to a wide range of knowledge areas and research institutions.

## Materials and Methods

This project was approved by the Ethics Committee of the Federal University of Rio Grande do Sul (CAAE 82423618.2.0000.5347). The study was performed using an online survey that was available for completion between April 22nd and May 25th, 2020. In this period, Brazilian day cares, schools, and universities had been closed due to the COVID-19 pandemic since approximately the second half of March.

### Sample

This survey was posted on social media and was e-mailed to universities and research centers based in Brazil. The snowball sampling technique was also used, where existing study subjects recruited future subjects from among their acquaintances. The survey took approximately 5 min to complete. Participants who failed to fully complete the questionnaire were excluded. The final sample was composed of 3,345 individuals, distributed throughout the country, of whom, the majority self-declared as White (75.9%), are women (68.4%) and are parents (70.7%).

### Survey Instrument

The questionnaire was specially developed to assess the impact of COVID-19 pandemic on the productivity of researchers of both genders with and without children. It consisted of 25 questions collecting information about the researchers’ demographics (country region, gender, and race), work setting (workplace closure, remote activities, online teaching) and children care (see a complete version of the questionnaire in the Supplementary Material). Productivity was assessed by the researchers’ self-reported ability to submit papers and meet deadlines during the pandemic period.

### Statistical Analysis

Data are presented as the percentage of respondents who were able to submit papers as planned and to meet deadlines related to grant/fellowship proposals and/or project/funding reports within each analyzed group. Statistical analysis to test for differences between groups (men and women; individuals with or without children, also stratified by the age of the youngest child; different races/ethnicities) was performed using a chi-squared test. Chi-squared analysis was performed in R using the chisq.test function. Pearson residual plots were generated with the corrplot package (version 0.84). Finally, pairwise comparisons between groups with statistically significant chi-squared tests were run with the chisq.multcomp function of the R package (version 0.9 - 77) using Bonferroni correction of *p*-values. The significance level was set at 0.05.

## Results

A detailed description of the survey respondents is provided in Table 1. The total sample size was 3,345 researchers, predominantly women (68.4%). Higher rates of female respondents in studies targeting university faculty members have been previously reported (Smith, 2008). In Brazil, women account for approximately 50% of the researcher population, according to the last Brazilian National Council for Scientific and Technological Development (CNPq) Census. The percentage of respondents from each region in Brazil followed the same pattern reported by the CNPq (6.3% from the North, 20.5% Northeast, 7.7% Center-west, 42.5% Southeast and 22.9% South), indicating that the sample of respondents is representative of the general academic population. Respondents self-identified as White (75.9%), 18.1% Black, 1.7% Asian, 0.2% Indigenous, and 4% did not inform the race/ethnicity. Considering the small percentage of Asians and Indigenous people, we only included in the analysis Black and White respondents. Most of the researchers have children: 33.8% have one, 30.2% have two, 5.8% have three, and 0.9% have four or more children.

**TABLE 1:** Characterization of the sample included in the study (3,345 respondents).

	General (%, *n*)	Male (%, *n*)	Female (%, *n*)
**Gender**		31.6 (1057)	68.4 (2288)
**Race/Ethnicity**^§^			
White	75.9 (2540)	73.8 (780)	76.9 (1760)
Black	18.1 (606)	18.9 (200)	17.7 (406)
Asian	1.7 (58)	1.1 (12)	2.0 (46)
Indigenous	0.2 (7)	0.2 (2)	0.2 (5)
ND*	4.0 (134)	5.9 (63)	3.1 (71)
**With children**	70.7 (2366)	67.6 (715)	72.2 (1651)
**Origin (Brazilian Region)^+^**			
North	6.2 (208)	6.3 (67)	6.1 (140)
Northeast	15.4 (515)	16.4 (173)	14.9 (342)
Center-west	8.7 (292)	10.0 (106)	8.1 (186)
Southeast	42.7 (1428)	38.9 (411)	44.4 (1016)
South	27.0 (904)	28.4 (300)	26.4 (604)
Academic Area^€^			
Agricultural Sciences	7.1 (237)	8.8 (93)	6.3 (144)
Biological Sciences	20.9 (698)	19.9 (210)	21.3 (488)
Engineering	5.2 (175)	6.9 (73)	4.5 (102)
Exact and Earth Sciences	17.6 (589)	26.7 (282)	13.4 (307)
Health Sciences	19.1 (639)	12.7 (134)	22.1 (505)
Humanities	12.7 (426)	9.7 (103)	14.1 (323)
Linguistics, Language and Arts	4.4 (149)	2.8 (30)	5.2 (119)
Multidisciplinary	3.4 (113)	3.2 (34)	3.4 (78)
Social Sciences	9.6 (320)	9.3 (98)	9.7 (222)

*General data are shown as percentages (%) of the total number of respondents.*

*Gender data are shown as percentages (%) of respondents of the same gender (male or female).*

*The total number of respondents from each category is presented as (n).*

^§^ Terminology follows the official Brazilian census and the Brazilian Institute of Geography and Statistics (IBGE). Race/ethnicity categories are based on a skin color continuum ranging from very fair to very dark skin. We adopt official IBGE categories in the questionnaires: branca (White), preta (Black), parda, amarela (Yellow: translated as Asian) and indigena (Indigeneous). In Brazil, there is a common distinction between people who identify as Black (dark-skin Black people) and parda (light-skin Black people). In all results presented in the report, the Black category refers to both IBGE categories (preta and parda) together.

*Prefer not to disclose.

*^+^The percentage of researchers for each region in Brazil, according to the last Brazilian National Council for Scientific and Technological Development (CNPq) Census, is 6.3% (North), 20.5% (Northeast), 7.7% (Center-west), 42.5% (Southeast), and 22.9% (South).*

*^€^Academic area nomenclature according to the CNPq classification. According to this, “Exact and Earth Sciences” include math, statistics, computer sciences, astronomy, physics, chemistry, geosciences, and oceanography.*

Productivity during the pandemic was assessed by analyzing self-reported data on manuscript submissions and the ability to meet deadlines. We also evaluated how scientists perceived the impact of the social isolation period on their productivity, as well as their perceptions of factors that interfered with their remote work routines. Regarding these perceptions, the researchers were asked if there were any factors in their current situation that impacted their remote work (e.g., childcare – routine care and/or homework assistance, children with disabilities, elderly care, and household chores).

### Manuscript Submissions During the Pandemic Period

**FIGURE 1:**
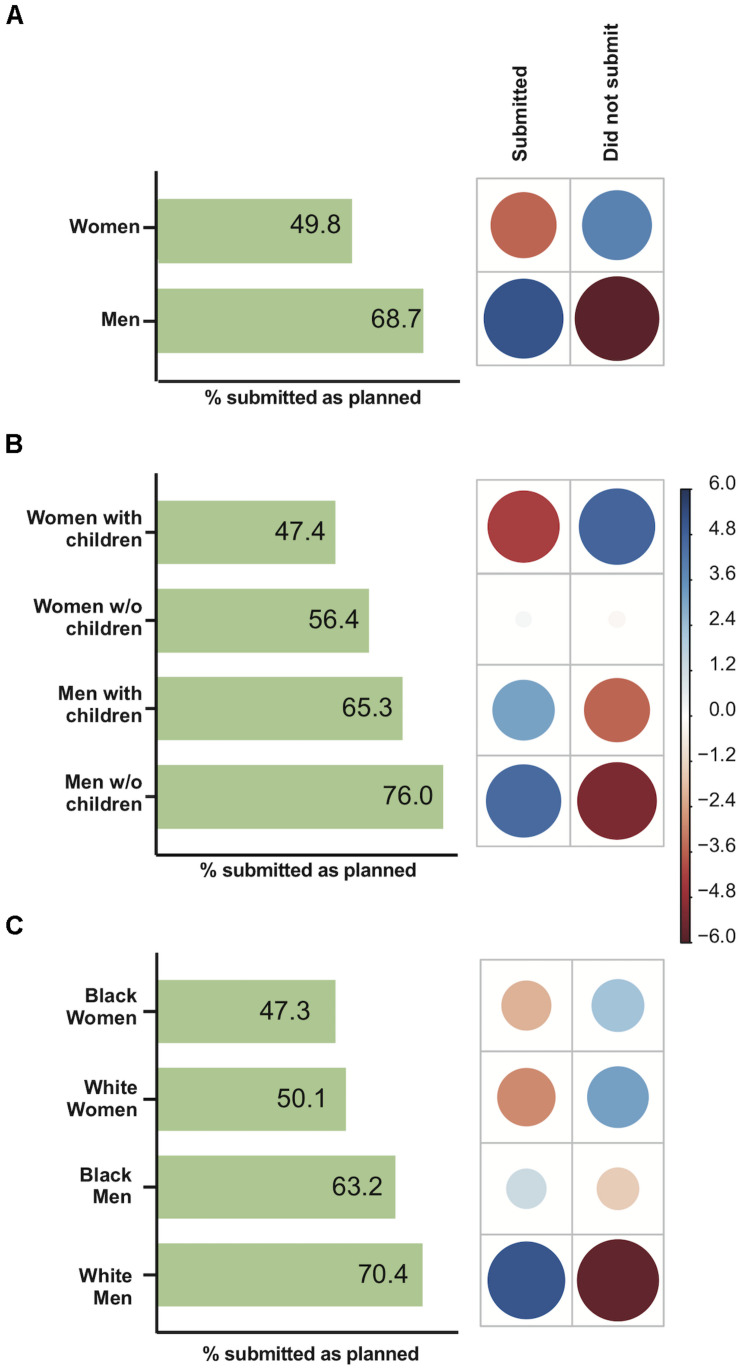
The impact of gender, parenthood and race on manuscript submissions during the COVID-19 pandemic. For each figure, the graph on the left-hand side represents the percentage of respondents who submitted manuscripts as planned, while on the right-hand side, the correlation plot shows Pearson’s chi-squared standardized residuals calculated for each group. Positive residuals (blue) indicate a positive correlation, whereas negative residuals (red) indicate a negative correlation. The size of the circle is proportional to the cell’s contribution to the χ^2^ score. **(A)** Gender effect on submissions. **(B)** Parenting effect on submissions. **(C)** Race effect on submissions.

Among the survey respondents, only 13.6% stated they did not have any manuscript being finalized for submission during the time that social isolation took place, so data on manuscript submission were analyzed excluding these respondents from the dataset. Manuscript submission among male academics was less affected by the pandemic circumstances than that among women (Figure 1A), with a significant difference between men and women (χ^2^ = 88.42, *P* < 0.0001). Positive associations were observed between women and the non-submission of manuscripts as well as between men and the submission of manuscripts (Figure 1A). There was a significant effect of parenthood on the submission of manuscripts (χ^2^ = 110.79, *P* < 0.0001) (Figure 1B). There was a positive association between women with children and the non-submission of manuscripts. However, no association was observed for women without children. The proportion of childless men who submitted manuscripts was higher than that of men with children (*P* < 0.01, Bonferroni *post hoc* test) (Figure 1B). Additionally, the proportion of childless women who submitted manuscripts was higher than that of women with children (*P* < 0.01, Bonferroni *post hoc* test) (Figure 1B). There was no overall race effect (Black vs. White researchers) on productivity during the pandemic period with respect to submissions (χ^2^ = 2.29, *p* = 0.1304) (Supplementary Figure 1), but there was a significant effect of race and gender on the submission of manuscripts (χ^2^ = 91.01, *P* < 0.0001) (Figure 1C). Positive associations were observed between White men and the submission of manuscripts as well as between both Black and White women and the non-submission of manuscripts (Figure 1C).

**FIGURE 2:**
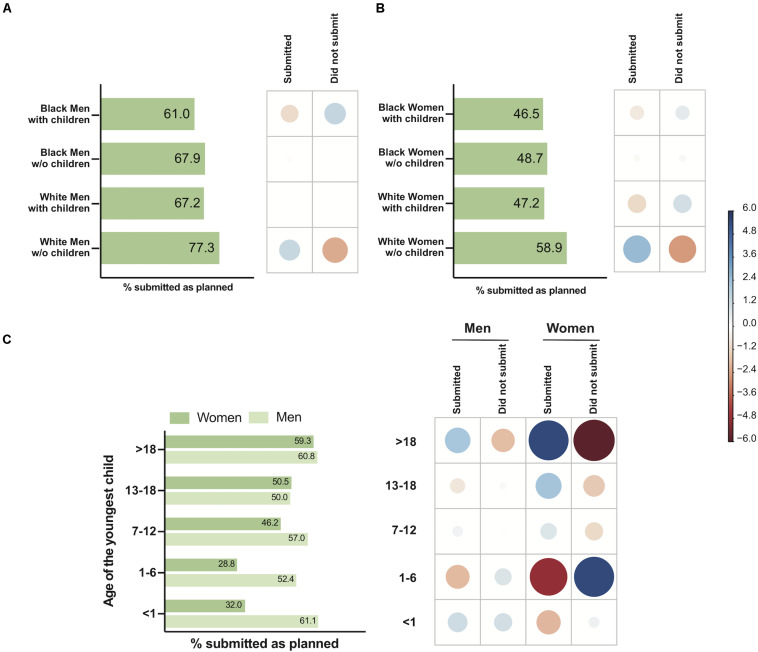
The influence of race, gender, parenthood, and youngest child’s age on the submission of manuscripts as planned during the COVID-19 pandemic. Left-hand panels show the percentage of men or women, Black or White, who submitted manuscripts as planned **(A,B)** and the percentage of men or women who submitted manuscripts as planned, according to the youngest child’s age **(C)**. The right-hand panels show the correlation plot with Pearson’s chi-squared standardized residuals calculated for each group. The color of the circles indicates a positive correlation (blue) or negative correlation (red), and the size of the circles is proportional to the cell’s contribution to the χ^2^ score. **(A)** Effect of race vs. parenthood for men on submissions. **(B)** Effect of race vs. parenthood for women on submissions. **(C)** Effect of the youngest child’s age vs. gender on submissions.

There was a significant difference among groups of men (Black with children, Black without children, White with children, White without children) with respect to the submission of manuscripts (χ^2^ = 10.93, *P* < 0.05) (Figure 2A). A negative association between White men without children and the non-submission of manuscripts was detected. The proportion of childless White men who submitted manuscripts was higher than that of White men with children (*P* < 0.05, Bonferroni *post hoc* test) (Figure 2A). Additionally, there was a significant difference among groups of women (Black with children, Black without children, White with children, White without children) with respect to the submission of manuscripts (χ^2^ = 16.43, *P* < 0.001) (Figure 2B). There was a positive association between White women without children and the submission of manuscripts. The proportion of childless White women who submitted manuscripts was higher than that of White women with children (*P* < 0.01, Bonferroni *post hoc* test) (Figure 2B). For Black women, there was no significant difference between the groups with and without children.

Children’s age was also associated with productivity. There was a significant difference between men and women depending on the age of their youngest child with respect to the submission of manuscripts (χ^2^ = 147.95, *P* < 0.0001) (Figure 2C). There was a negative association between women whose youngest child ranged from 1 to 6 years old and the submission of manuscripts. The proportion of this group’s submissions was lower than that of men with children of the same age (*P* < 0.001, Bonferroni *post hoc* test) (Figure 2C). Additionally, the proportion of submissions observed for men whose youngest child’s age ranged from 7 to 12 were higher than that observed for women with children of the same ages (*P* < 0.001, Bonferroni *post hoc* test) (Figure 2C).

### Ability to Meet Deadlines

**FIGURE 3:**
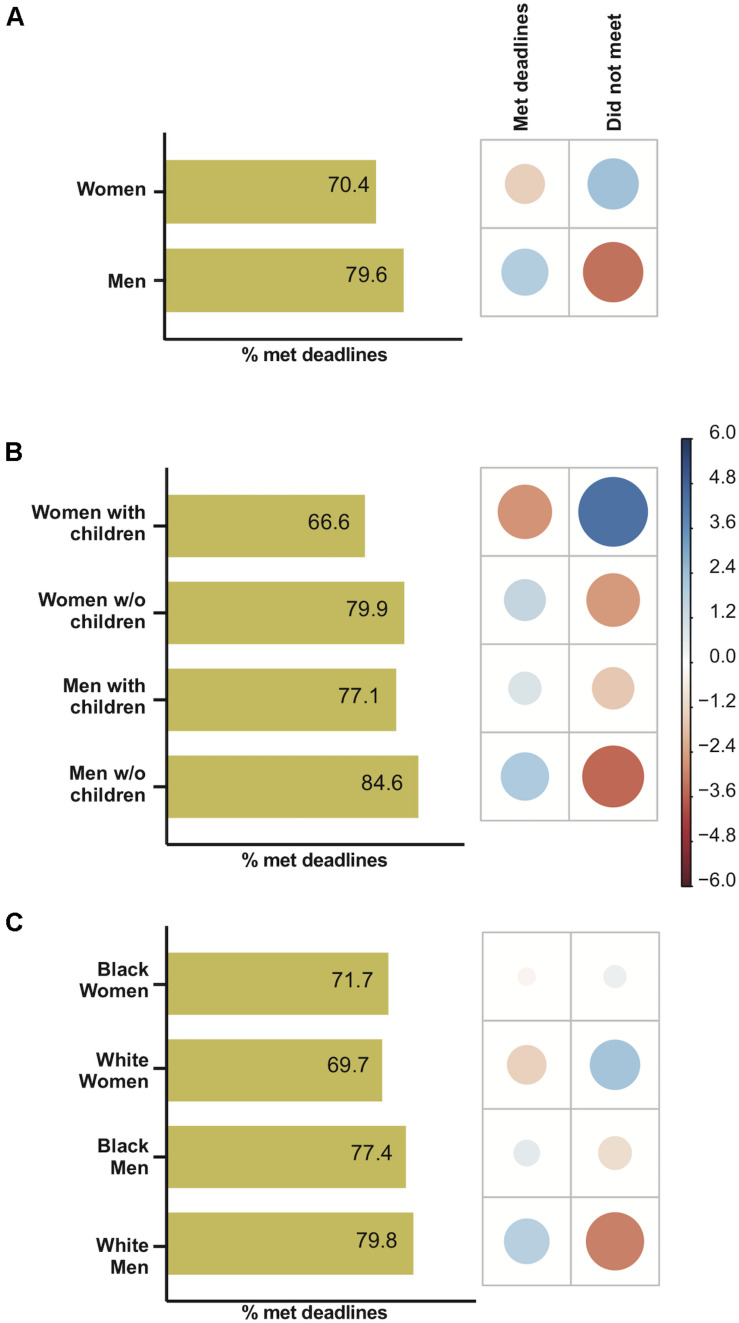
The impact of gender, parenthood and race on meeting deadlines during the COVID-19 pandemic. For each figure, the graph on the left-hand side represents the percentage of respondents who submitted manuscripts as planned, while on the right-hand side, the correlation plot shows Pearson’s chi-squared standardized residuals calculated for each group. Positive residuals (blue) indicate a positive correlation, whereas negative residuals (red) indicate a negative correlation. The size of the circle is proportional to the cell’s contribution to the χ^2^ score. **(A)** Gender effect on meeting deadlines. **(B)** Parenthood effect on meeting deadlines. **(C)** Race effect on meeting deadlines.

The respondents were asked whether the pandemic situation impacted how they met deadlines. There was a significant difference between men and women (χ^2^ = 21.73, *P* < 0.0001) regarding the ability to meet deadlines during the pandemic (Figure 3A). Positive associations between women and the failure to meet deadlines and between men and the ability to successfully meet deadlines were observed (Figure 3A). Parenthood was significantly associated with the ability to meet deadlines (χ^2^ = 55.33, *P* < 0.0001) (Figure 3B). Positive associations between women with children and the failure to meet deadlines and between men without children and the ability to successfully meet deadlines were detected. There was a significant difference (*P* < 0.0001, Bonferroni *post hoc* comparison) between the proportions of women and men with children who met deadlines (Figure 3B). Moreover, the proportion of women without children who met deadlines was higher than that of women with children (*P* < 0.0001 Bonferroni *post hoc* comparison) (Figure 3B). There was no overall correlation of race (Black vs. White researchers) with productivity during the pandemic period in relation to meeting deadlines (χ^2^ = 0.06, *p* = 0.7956) (Supplementary Figure 1). There was a significant association of race and gender for meeting deadlines (χ^2^ = 21.39, *P* < 0.0001) (Figure 3C). A significant difference was observed between the proportions of White men and White women who met deadlines (*P* < 0.0001, Bonferroni *post hoc* comparison).

**FIGURE 4:**
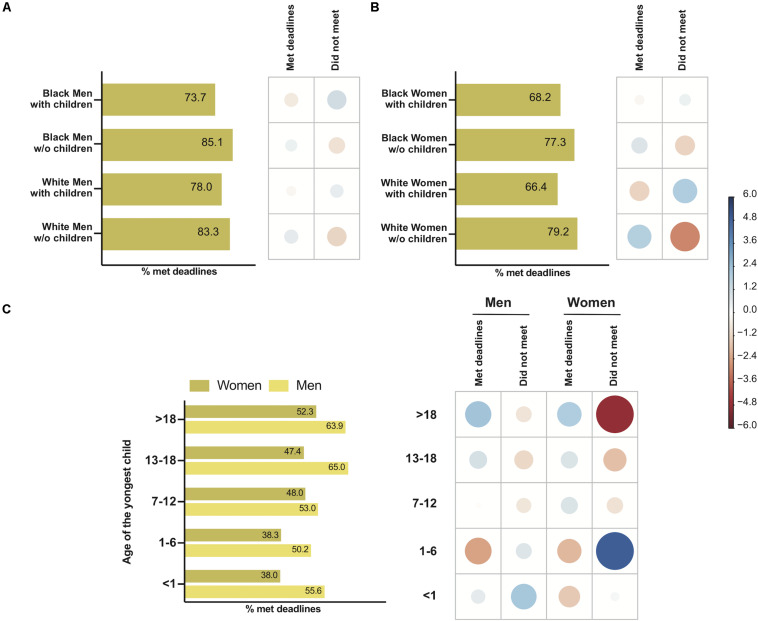
The influence of race, gender, parenthood, and youngest child’s age on meeting deadlines during the COVID-19 pandemic. Left-hand panels show the percentage of men or women, Black or White, who were able to meet deadlines **(A,B)** and the percentage of men or women who met deadlines, according to the youngest child’s age **(C)**. The right-hand panels show the correlation plot with Pearson’s chi-squared standardized residuals calculated for each group. The color of the circles indicates a positive correlation (blue) or negative correlation (red), and the size of the circles is proportional to the cell’s contribution to the χ^2^ score. **(A)** Effect of race vs. parenting for men on meeting deadlines. **(B)** Effect of race vs. parenthood for women on meeting deadlines. **(C)** Effect of the youngest child age vs. gender on meeting deadlines.

There was no significant difference between groups (Black with children, Black without children, White with children, White without children) among men (χ^2^ = 5.15, *P* = 0.1611) (Figure 4A), but there was a significant difference among groups of women (Black with children, Black without children, White with children, White without children) with respect to meeting deadlines (χ^2^ = 20.62, *P* < 0.01) (Figure 4B). There was a negative association between White women without children and the failure to meet deadlines. The proportion of childless White women who met deadlines was higher than that of White women with children (*P* < 0.001, Bonferroni *post hoc* test) (Figure 4B). There was no significant difference between the proportions of Black women without children and Black women with children who met deadlines (Bonferroni *post hoc* comparison).

Children’s age also influenced the ability to meet deadlines, as observed for manuscript submission. There was a significant difference between men and women depending on the age of their youngest child (χ^2^ = 83.37, *P* < 0.0001) (Figure 4C). The proportion of women with children who were able to meet deadlines was lower than men with children that met the deadline, regardless of the age of the youngest child (*P* < 0.01 for all comparisons, Bonferroni *post hoc* test) (Figure 4C).

### Impact of Remote Work on Productivity

Respondents were asked to evaluate how the period of institution closures and the imposed adaptation to remote work had affected their productivity (indicating whether the impact was negative, non-existent or positive). The intersection between race, gender and parenthood was analyzed considering how respondents self-reported the impact of remote work on their productivity. The majority (69.4%) of respondents stated that they had felt a negative impact on their productivity, while only 16.2 and 14.4% reported positive or no impacts, respectively.

**FIGURE 5:**
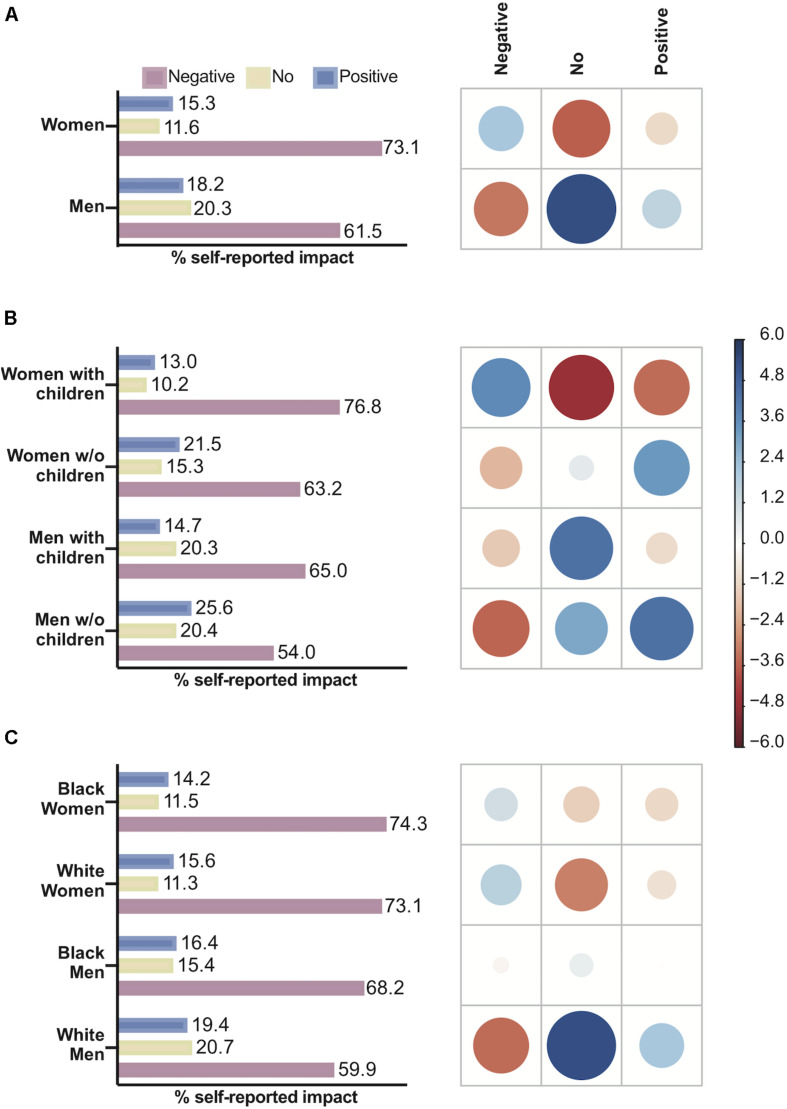
The effect of gender, parenthood and race on the self-reported impact of the remote work regimen on productivity. For each figure, the graph on the left-hand side represents the percentage of respondents who reported negative, no or positive impacts, while on the right-hand side, the correlation plot shows Pearson’s chi-squared standardized residuals calculated for each group. Positive residuals (blue) indicate a positive correlation, whereas negative residuals (red) indicate a negative correlation. The size of the circle is proportional to the cell’s contribution to the χ^2^ score. **(A)** Gender effect on self-reported impact. **(B)** Parenthood effect on self-reported impact. **(C)** Race effect on self-reported impact.

There was a significant difference between the way men and women perceived the impact of the pandemic on their productivity during the social isolation period (χ^2^ = 61.06, *P* < 0.0001) (Figure 5A). We observed a statistically significant positive association of men and the perception of no impact in productivity, and between women and a negative impact in productivity. There was a significant difference between men and women who perceived a positive impact (*P* < 0.001, Bonferroni *post hoc* test). Parenthood influenced the way respondents perceived the impact of remote work on their productivity (χ^2^ = 127.56, *P* < 0.0001) (Figure 5B), especially for women. There was a positive association between women with children and a negative impact (*P* < 0.0001), but this association was not observed for men with children. Race was also related to the way respondents perceived the impact of remote work on their productivity (χ^2^ = 62.63, *P* < 0.0001) (Figure 5C). White men reported a negative impact less frequently than Black men and Black and White women (*P* < 0.001, Bonferroni *post hoc* test for all comparisons).

**FIGURE 6:**
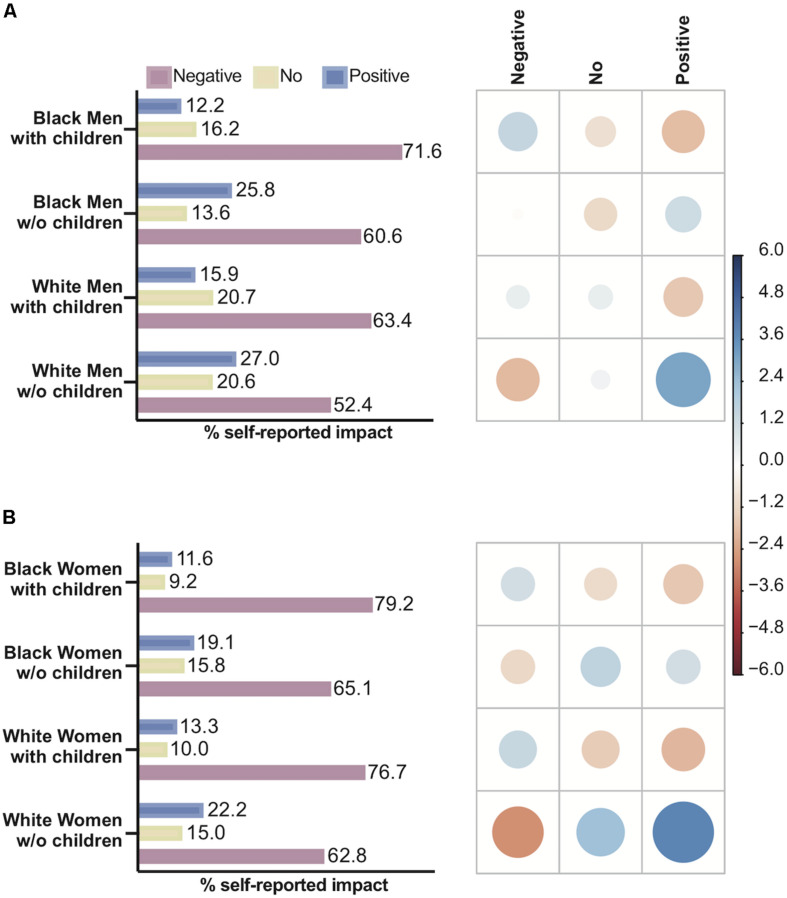
The influence of race, gender and parenthood on the self-reported impact of remote work regimen on productivity. Left-hand panels show the percentage of men or women, Black or White, who reported negative, no or positive impacts. The right-hand panels show the correlation plot with Pearson’s chi-squared standardized residuals calculated for each group. The color of the circles indicates a positive correlation (blue) or negative correlation (red), and the size of the circles is proportional to the cell’s contribution to the χ^2^ score. **(A)** Effect of race vs. parenthood for men on self-reported impact. **(B)** Effect of race vs. parenthood for women on self-reported impact.

Parenthood influenced the self-reported impact of the pandemic for White and Black men (χ^2^ = 26.15, *P* < 0.0001) (Figure 6A) and for White and Black women (χ^2^ = x 46.65, *P* < 0.0001) (Figure 6B). When the analysis considered all intersections between gender, parenthood and race, there was a significant difference between White and Black men with children who felt a negative impact and White and Black men without children, respectively (*P* < 0.001, Bonferroni *post hoc* test). There was a positive association between White men without children and a positive impact on productivity, and this association was weaker for Black men without children. There was a significant difference between White and Black women with children and White and Black women without children (*P* < 0.001, Bonferroni *post hoc* test), but there was no difference between Black and White mothers with respect to the impact on their productivity.

### Respondents’ Perception of the Factors Impacting Their Remote Work Routines During the Pandemic

The respondents were asked to list any factors in their current situation that impacted their productivity during remote work. Among respondents with children, domestic labor was perceived as a factor influencing remote work for 88.7% of Black mothers, 86.1% of White mothers, 78.4% of Black fathers and 70.0% of White fathers. The routine care of children was more commonly a factor listed by women (80.2 and 80.1% of Black and White mothers, respectively) than by men (69.6 and 61.5% of Black and White fathers, respectively). All groups listed children’s school activities as a factor perceived as influencing remote work: Black mothers (48.8%), White mothers (46.1%), Black fathers (43.2%) and White fathers (39.6%). The care of family members (other than their own children) was listed by 18.8 and 15.5% of Black and White mothers, respectively, and by 9.5 and 12.5% of Black and White fathers, respectively. For all groups of respondents with children, mental health issues were uncommonly (less than 1.7%) perceived as influencing their remote work at the time the survey was conducted.

Among childless respondents, 76.3% of Black women and 71.9% of White women perceived domestic labor as influencing their remote work routines, compared to 62.1 and 65.2% of Black and White men, respectively. The care of family members was more commonly among the factors listed by women (32.2 and 29.4% for Black and White women, respectively) than by men (22.7 and 22.5% for Black and White men, respectively). Mental health issues were listed by 7.9% of Black women, 9.4% of White women, and 4.5% of both Black and White men.

## Discussion

Our results suggest that gender, parenthood and race are associated with the ability to submit manuscripts and to meet deadlines during the pandemic period. Nevertheless, not all scientists are affected in the same way: White mothers and Black females, regardless of whether they are mothers, are the groups taking the strongest hit in academia. Our study is the first to provide conclusive data on the main forces – race and motherhood – driving the productivity imbalance in science during the pandemic. Our results for the Brazilian context echo those of studies based on the US context showing that working mothers, including those in academia, might be disproportionately affected by the COVID-19 crisis (Alon et al., 2020; Wenham et al., 2020). This exacerbated disparity during the pandemic reflects the historical inequality between the careers of men and women.

Data from before the pandemic indicate that women spend significantly more time on household labor and chores than men (Bianchi et al., 2012), including women in scientific careers (Gupta et al., 2005; Jolly et al., 2014) in diverse cultures of India, Germany and the United States. On average, women spend two more hours (5.7 h) each day than men (3.6 h) on caretaking, cleaning, cooking, and doing other domestic work in the United States (Hess et al., 2020). In Brazil, men spend 10.5 h per week on similar activities, caring for children or doing other chores, while women devote 18.1 h per week (nearly 73% more than men) on these tasks (IBGE, 2018). This unbalanced division of domestic tasks between men and women has a huge impact on women’s careers, including employment and economic costs, as many caregivers cut back on the time spent in paid work (Lilly et al., 2007). The reduced time dedicated to the paid workforce leads to fewer opportunities for advancement, since a “successful position” in leadership roles often involves working long hours. These more limited opportunities for promotion can contribute to the gender gap, especially at the height of women’s careers. Considering the maternity penalty in particular, women can suffer a decrease in work productivity after the birth of their children in different countries and cultures (Gallen, 2018; Machado et al., 2019). As a result, an increase in the gender gap after motherhood occurs in many fields (Angelov et al., 2016; Hardoy et al., 2017; Kleven et al., 2019; Collins et al., 2020), including academia, where mothers in the United States, spend 8.5 more hours per week on parenting or domestic tasks and less time on research than fathers (Mason and Goulden, 2004; Jolly et al., 2014). Women academics in the United States. also take on tasks such as waking up during the night and staying at home to care for a sick child (Rhoads and Rhoads, 2012). This asymmetrical division of parenting and domestic tasks can be reflected in a decrease in the number of annual scientific publications by Brazilian academic women (Machado et al., 2019), thus affecting the career progression of mothers in academia. Other significant barriers to women’s progress include gender stereotypes and implicit gender bias, which are invisible and powerful forces preventing women from advancing in their careers. The stereotype that women are less competent and less hirable than men (Moss-Racusin et al., 2012; Reuben et al., 2014; Eaton et al., 2020) creates unfair disadvantages for women scientists, including lower salaries and less career mentoring (Moss-Racusin et al., 2012). For instance, among articles published in Nature research journals, only 18.1% have women as senior authors (last authorship), and the higher the journal’s impact index is, the smaller the number of women listed as the principal author (Bendels et al., 2018). Importantly, however, the number of articles published with women listed as the first author increases when articles are reviewed anonymously (Budden et al., 2008). In terms of obtaining research funding, the effects of the implicit gender bias against women are also substantial. Women in Sweden need to author twice as many publications to obtain the same scientific competence score as men to obtain a postdoctoral position (Wenneras and Wold, 1997). A study revealed that men obtain more funding renewals than women considering funding provided by the National Institutes of Health in the United States (Pohlhaus et al., 2011).

During the COVID-19 pandemic, several factors that have historically promoted gender inequalities, such as those mentioned above, appear to have increased. For instance, Andersen et al. (2020) argue that the school closures and distancing requirements that have disrupted both work and family life for many people may not have influenced men and women researchers equally. Similarly, female academics based on the United States and Europe “reported larger declines in the time they could devote to research than their male colleagues during the pandemic” (Myers et al., 2020), which, according to the authors, will likely continue to evolve and have longer-term impacts on science. In trying to explain the gender gap found in the pandemic’s effects on publishing, Viglione (2020) says that female faculty usually carry more teaching responsibilities, so the sudden shift to online teaching has affected them disproportionately. Malisch et al. (2020) suggest that the transition to remote teaching, changes in grading systems, the loss of access to research resources, and shifts in household labor, childcare and eldercare are ways in which COVID-19 is amplifying known barriers to women’s career advancement (Malisch et al., 2020). Early career bias has also been proposed as an explanation for the lower paper submission rates of women in academia during this period (Andersen et al., 2020; Viglione, 2020); the early career period aligns with the reproductive age of these women (Morgan, 2015). Not surprisingly, our results showed that children’s age had an impact on Brazilian academic mothers’ productivity during the pandemic. Young children require much more attention and care, and parents face additional demands related to having time to homeschool children during the social isolation period. Indeed, studies carried out in the United States and Europe showed mothers with young children reported a reduction in work hours (Collins et al., 2020; Myers et al., 2020). The smaller number of hours dedicated to research likely reduces the paper submission rate among women, which we have in fact demonstrated. Additionally, as stated by Malisch et al. (2020), the burden is even heavier for women who face intersecting systems of oppression, for example, ethnicity and race.

Gender inequality intersects with the racial profile of academics. Indeed, Black women are greatly underrepresented in science in the United States (McGee and Bentley, 2017). Our data confirmed that it also occurs in Brazil, showing that Black female academics, regardless of the motherhood factor, are the group most affected by the pandemic circumstances. Interestingly, the productivity of White women without children was not affected as much as that of Black women without children, but in both groups, the effect was higher than that observed for childless men, regardless of race. One possible explanation for this finding is that women, particularly Black women, have less social network support than men, which can negatively influence their career trajectory (Feeney and Bernal, 2010; Collins and Steffen-Fluhr, 2019). Black women frequently experience isolation and a sense of “not belonging” (Ong et al., 2018). As proposed by Smith et al. (2007), feelings of isolation and not belonging can elicit “racial battle fatigue” in Black women, i.e., the “cumulative result of a natural race-related stress response to distressing mental and emotional conditions” that adversely impacts the health and accomplishments of Black people (Smith et al., 2007; Corbin et al., 2018). Black women eschew academic careers altogether or exit the academy prior to tenure decisions because they experience social isolation, an unwelcoming environment, bias, and hostility (Trower and Chait, 2002). In academia, networks play a direct role in career success through employment, publication, and conference opportunities, and they can also have less direct impacts, such as by positioning researchers closer to burgeoning research trends, which allows them to work with the most recent data (Heffernan, 2020). The reasons behind the pronounced impact of the pandemic on Black female researchers’ productivity, regardless of motherhood, are still debatable; however, the lack of professional networks due to structural racism might play a central role since the challenges of networking can be exacerbated during the pandemic. Working from home poses unique authenticity challenges for Black people, especially Black women, whose colleagues now have windows into their personal lives that could amplify portrayals of them as the “other.” This is because “professionalism” is coded by white middle-/upper-social-class standards and Black workers are disproportionately affected by judgments of professionalism and cultural fit (Roberts and McCluney, 2020). Besides, Black patients still die far more frequently than White patients in Brazil (Peres et al., 2021), and Black women are overloaded on the responsibilities for extended family members, including financial responsibilities in the United States (Black et al., 2009).

Although we were able to confirm the association between the COVID-19 pandemic and the lower productivity of women scientists observed in previous studies (Andersen et al., 2020; Myers et al., 2020; Viglione, 2020), our study has some drawbacks that need to be acknowledged. The first concerns the snowball methodology used, which has a sample bias, as study subjects recruit future subjects among their acquaintances. This limitation does not prevent the use of the snowball methodology for a considerable number of studies (see Noy, 2008; Christopoulos, 2009); however, to minimize this problem, we sent emails to all Brazilian graduate programs registered in the Coordination for the Improvement of Higher Education Personnel (CAPES) database, requesting that they share the invitation to participate in the survey with researchers. With these efforts, we obtained a good fit between the number of responses to our questionnaire and the distribution of scientists from different regions of Brazil (see the section “Results”). Therefore, we believe that our results are, to a certain extent, representative of the geographic regions of Brazil. The second limitation is the bias generated by the number of women respondents, who comprised close to 70% of our sample. This bias seems to be a general effect in this type of study, since women are more responsive to online research, regardless of the purpose of the study (Smith, 2008). Additionally, we obtained a good number of responses for all groups analyzed, since our sample size was relatively large.

In summary, our findings revealed that female academics, especially Black females and mothers (regardless of race), are absorbing the greatest costs of the pandemic. This fact could lead to an unprecedented increase in both gender and race gaps in science. The situation we are facing during the pandemic demands actions from our institutions, and academia should foster a discussion about policies to benefit Black scientists and academics with families in the post-pandemic context.

The short-run challenges posed by the crisis are severe, especially for single mothers and other families with a lack of ability to combine work with caring for children at home (Alon et al., 2020). Ensuring that women’s academic output is not disproportionately affected by COVID-19 might safeguard women’s career trajectories (Gabster et al., 2020) and affect the overall science landscape. Our study strongly recommends the implementation of policies and actions to mitigate this reality, such as those proposed by Cardel et al. (2020) and Hipólito et al. (2020). The international academic community needs flexibility in institutional policies from research institutions and funding agencies, such as the postponement of deadlines for grant proposals and reports. This is especially important in cases where researchers had caregiving responsibilities during the pandemic. Extending deadlines does not require much investment in terms of public funding and can have a positive impact in allowing people with reduced time dedicated to work to still apply and compete for research grants. Furthermore, funding agencies should consider creating grants designed to benefit Black scientists and academics with families. Actions such as these would reward the most underrepresented and vulnerable groups. It is important to avoid an increase in gender and racial differences after the pandemic. Immediate actions to mitigate the weight women are carrying during the pandemic period include allowing flexible working arrangements, where administrative activities and teaching schedules are carried out by colleagues with more flexibility, and, where possible, not holding meetings during times that conflict with homeschooling hours. Another important point is to create an infrastructure for family care in academic spaces while schools and daycare centers remain closed. This is an issue that should be openly discussed within departments, and collective solutions should be built to reduce the foreseen amplification of the gender gap. Additionally, in a broader sense, evaluations of manuscripts for publication and career assessments should prioritize race and gender equity, especially when the timeframe for evaluations includes 2020 and 2021. The COVID-19 CV Matrix proposed by Arora et al. (2020) is a framework that can enable faculty members to account for their contributions, disruptions, and caregiving responsibilities and can provide promotion and tenure committees a better way to fairly evaluate faculty members during the pandemic period. In times of growing compassion, we invite the entire scientific community to make science more diverse and fairer after the pandemic.

## Data Availability Statement

The raw data supporting the conclusions of this article will be made available by the authors, without undue reservation.

## Ethics Statement

This project was approved by the Ethics Committee of the Federal University of Rio Grande do Sul (CAAE 82423618.2.0000.5347). Written informed consent for participation was not required for this study in accordance with the national legislation and the institutional requirements.

## Author Contributions

FS, LK, EZ, FR, RS, AN, IS, PM-C, AT, FW, FKR, CI, AS, and LO: conceptualization. FS, LK, EZ, FR, RS, ZL, EL, AN, IS, PM-C, AT, FW, FKR, CI, and AS: data curation. FS, LK, EZ, FR, RS, EL, AN, IS, PM-C, AT, FW, FKR, CI, AS, CS, and LO: formal analysis. EZ, IS, PM-C, CS, and LO: funding acquisition. FS, LK, EZ, FR, RS, ZL, EL, AN, IS, PM-C, AT, FW, FKR, CI, AS, CS, and LO: investigation and methodology. FS, LK, EZ, FR, RS, ZL, EL, AN, IS, PM, AT, FW, FKR, CI, AS, CS, and LO: project administration and writing – review and editing. LK and CS: resources. FS, LK, EZ, FR, RS, and LO: supervision. FS, LK, EZ, FR, RS, EL, AN, PM, AT, FW, FKR, CS, and LO: visualization. FS, LK, EZ, FR, RS, ZL, EL, AN, IS, PM, AT, FW, FKR, CI, and LO: writing – original draft. All authors contributed to the article and approved the submitted version.

## Conflict of Interest

The authors declare that the research was conducted in the absence of any commercial or financial relationships that could be construed as a potential conflict of interest.

## References

[B1] Acker J. (2006). Inequality regimes: gender, class, and race in organizations. *Gend. Soc.* 20 441–464. 10.1177/0891243206289499

[B2] Alon T. Doepke M. Olmstead-Rumsey J. Tertilt M. (2020). *The Impact of COVID-19 on Gender Equality. CRC TR 224 Discussion Paper Series crctr224_2020_163.* Mannheim: University of Bonn and University of Mannheim.

[B3] Andersen J. P. Nielsen M. W. Simone N. L. Lewiss R. E. Jagsi R. (2020). COVID-19 medical papers have fewer women first authors than expected. *eLife* 9:e58807. 10.7554/elife.58807 32538780 PMC7304994

[B4] Angelov N. Johansson P. Lindahl E. (2016). Parenthood and the gender gap in pay. *J. Labor. Econ.* 34 545–579. 10.1086/684851

[B5] Antecol H. Bedard K. Stearns J. (2018). Equal but inequitable: who benefits from gender-neutral tenure clock stopping policies? *Am. Econ. Rev.* 108 2420–2441. 10.1257/aer.20160613

[B6] Arora V. M. Wray C. M. O’Glasser A. Y. Shapiro M. Jain S. (2020). Using the curriculum vitae to promote gender equity during the COVID-19 pandemic. *Proc. Natl. Acad. Sci.U. S. A.* 117 24032–24032. 10.1073/pnas.2012969117 32943540 PMC7533702

[B7] Bendels M. H. K. Müller R. Brueggmann D. Groneberg D. A. (2018). Gender disparities in high-quality research revealed by nature index journals. *PLoS One* 13:e0189136. 10.1371/journal.pone.0189136 29293499 PMC5749692

[B8] Bianchi S. M. Sayer L. C. Milkie M. A. Robinson J. P. (2012). Housework: who did, does or will do it, and how much does it matter? *Soc. Forces* 91 55–63. 10.1093/sf/sos120 25429165 PMC4242525

[B9] Black A. R. Murry V. M. Cutrona C. E. Chen Y. (2009). Multiple roles, multiple lives: the protective effects of role responsibilities on the health functioning of African American mothers. *Women Health* 49 144–163. 10.1080/03630240902915051 19533507 PMC2743987

[B10] Britton D. M. (2014). Do babies matter? gender and family in the ivory tower by mary ann mason, nicholas h. wolfinger, and marc goulden. *Am. J. Sociol.* 120 988–990. 10.1086/678475

[B11] Brooks C. Fenton E. M. Walker J. T. (2014). Gender and the evaluation of research. *Res. Policy* 43 990–1001. 10.1016/j.respol.2013.12.005

[B12] Budden A. Tregenza T. Aarssen L. W. Koricheva J. Leimu R. Lortie C. J. (2008). Double-blind review favours increased representation of female authors. *Trends Ecol. and Evol.* 23 4–6. 10.1016/j.tree.2007.07.008 17963996

[B13] Cardel M. I. Dean N. Montoya-Williams D. (2020). Preventing a secondary epidemic of lost early career scientists. Effects of covid-19 pandemic on women with children. *Ann. Am. Thorac. Soc.* 17 1366–1370. 10.1513/AnnalsATS.202006-589IP 32667850 PMC7640734

[B14] Carli L. L. Alawa L. Lee Y. Zhao B. Kim E. (2016). Stereotypes about gender and science: women ≠ scientists. *Psychol. Women Q*. 40 244–260. 10.1177/0361684315622645

[B15] Cech E. A. Blair-Loy M. (2019). The changing career trajectories of new parents in stem. *Proc. Natl. Acad. Sci. U. S. A.* 116 4182–4187. 10.1073/pnas.1810862116 30782835 PMC6410805

[B16] Christopoulos D. (2009). “Peer esteem snowballing: a methodology for expert surveys,” in *Proceedings of the Eurostat Conference for New Techniques and Technologies for Statistics* (Bristol: University of the West of England), 171–179

[B17] Collins C. Landivar L. C. Ruppanner L. Scarborough W. J. (2020). Covid-19 and the gender gap in work hours. *Gend. Work. Organ.* 28 101–112. 10.1111/gwao.12506 32837019 PMC7361447

[B18] Collins R. Steffen-Fluhr N. (2019). Hidden patterns: using social network analysis to track career trajectories of women STEM faculty. *Equal. Divers. Incl.* 38 265–282. 10.1108/edi-09-2017-0183

[B19] Corbin N. A. Smith W. A. Garcia J. R. (2018). Trapped between justified anger and being the strong black woman: black college women coping with racial battle fatigue at historically and predominantly white institutions. *Int. J. Qual. Stud. Educ.* 31 626–643. 10.1080/09518398.2018.1468045

[B20] da Silva J. (2010). Doutoras professoras negras: O que nos dizem os indicadores oficiais. *Perspectiva* 28 19–36. 10.5007/2175-795X.2010v28n1p19

[B21] Eaton A. A. Saunders J. F. Jacobson R. K. West K. (2020). How gender and race stereotypes impact the advancement of scholars in STEM: professors’ biased evaluations of physics and biology post-doctoral candidates. *Sex Roles* 82 127–141. 10.1007/s11199-019-01052-w

[B22] Feeney M. K. Bernal M. (2010). Women in STEM networks: who seeks advice and support from women scientists? *Scientometrics* 85 767–790. 10.1007/s11192-010-0256-y

[B23] Frietsch R. Haller I. Funken-Vrohlings M. Grupp H. (2009). Gender-specific patterns in patenting and publishing. *Res. Policy* 38 590–599. 10.1016/j.respol.2009.01.019

[B24] Gabster B. P. van Daalen K. Dhatt R. Barry M. (2020). Challenges for the female academic during the COVID-19 pandemic. *Lancet* 395 1968–1970. 10.1016/s0140-6736(20)31412-4 PMC7302767 32563275

[B25] Gallen Y. (2018). *Motherhood and the Gender Productivity Gap. Working Papers 2018-091.* Chicago, IL: Human Capital and Economic Opportunity Working Group.

[B26] Garbe A. Ogurlu U. Logan N. Cook P. (2020). Parents’ experiences with remote education during covid-19 school closures. *Am. J. Qual. Res.* 4 45–65. 10.29333/ajqr/8471

[B27] Gaston N. (2015). *Why is Science Sexist?* Vol. 34. Wellington: BWB Texts Book.

[B28] Gupta N. Kemelgor C. Fuchs S. Etzkowitz H. (2005). Triple burden on women in science: a cross-cultural analysis. *Curr.Sci.* 89 1382–1386

[B29] Gutiérrez y Muhs G. Niemann Y. F. Gonzalez C. G. Harris A. P. (2012). *Presumed Incompetent: The Intersections of Race and Class for Women in Academia.* Utah: Utah State University Press.

[B30] Hardoy I. Schøne P. Østbakken K. M. (2017). Children and the gender gap in management. *Labour Econ.* 47 124–137. 10.1016/j.labeco.2017.05

[B31] Heffernan T. (2020). Academic networks and career trajectory: ‘there’s no career in academia without networks’. *High. Educ. Res. and Dev.* 1–14. 10.1080/07294360.2020.1799948

[B32] Herman C. Lewis S. (2012). Entitled to a sustainable career? motherhood in science, engineering, and technology. *J. Soc. Issues* 68 767–789. 10.1111/j.1540-4560.2012.01775.x

[B33] Hess C. Ahmed T. Hayes J. (2020). *Providing Unpaid Household and Care Work in the United States: Uncovering Inequality. Job Quality and Income Security.* Washington, D.C: Institute for Women’s Policy Research.

[B34] Hipólito J. Diele-Viegas L. M. Cordeiro T. E. F. Sales L. P. Medeiros A. Deegan K.R. (2020). Unwrapping the long-term impacts of COVID-19 pandemic on Brazilian academic mothers: the urgency of short, medium, and long-term measures. *An. Acad. Bras. Ciênc.* 92:e20201292. 10.1590/0001-3765202020201292 33146238

[B35] Hofstra B. Kulkarni V. V. Galvez S. M. N. He B. Jurafsky D. McFarland D. A. (2020). The diversity–innovation paradox in science. *Proc. Natl. Acad. Sci. U. S. A.* 117 9284–9291. 32291335 10.1073/pnas.1915378117 PMC7196824

[B36] Hunt J. Garant J.-P. Herman H. Munroe D. J. (2013). Why are women underrepresented amongst patentees? *Res. Policy* 42 831–843. 10.1016/j.respol.2012.11.004

[B37] IBGE (2018). *Gender Statistics: Household Chores Affect Insertion of Women in Labor Market. Tech. Rep.* Rio de Janeiro: Brazilian Institute of Geography and Statistics.

[B38] Isgro K. Castañeda M. (2015). Mothers in US academia: insights from lived experiences. *Womens Stud. Int. Forum* 53 174–181. 10.1016/j.wsif.2014.12.002

[B39] James A. Chisnall R. Plank M. J. (2019). Gender and societies: a grassroots approach to women in science. *Royal Soc. Open Sci.* 6:190633. 10.1098/rsos.190633 31598298 PMC6774970

[B40] Jolly S. Griffith K.A. DeCastro R. Stewart A. Ubel P. Jagsi R. (2014). Gender differences in time spent on parenting and domestic responsibilities by high-achieving young physician-researchers. *Ann. Intern. Med.* 160 344–353. 10.7326/m13-0974 24737273 PMC4131769

[B41] Kleven H. Landais C. Søgaard J. E. (2019). Children and gender inequality: evidence from denmark. *Am. Econ. J. Appl. Econ.* 11 181–209. 10.1257/app.20180010

[B42] Krefting L. A. (2003). Intertwined discourses of merit and gender: evidence from academic employment in the USA. *Gend. Work Organ.* 10 260–278. 10.1111/1468-0432.t01-1-00014

[B43] Kyvik S. (1990). Motherhood and scientific productivity. *Soc. Stud. Sci.* 20 149–160. 10.1177/030631290020001005

[B44] Langin K. (2019). Women of color face double dose of bias. *Science* 364 921–922. 10.1126/science.364.6444.921 31171676

[B45] Lerchenmueller M. J. Sorenson O. (2018). The gender gap in early career transitions in the life sciences. *Res. Policy* 47 1007–1017. 10.1016/j.respol.2018.02.009

[B46] Lilly M. B. Laporte A. Coyte P. C. (2007). Labor market work and home cares unpaid caregivers: a systematic review of labor force participation rates, predictors of labor market withdrawal, and hours of work. *Milbank Q.* 85 641–690. 10.1111/j.1468-0009.2007.00504.x 18070333 PMC2690351

[B47] Lunnemann P. Jensen L. Mogens H. Jauffred L. (2019). Gender bias in nobel prizes. *Palgrave Commun.* 5:46. 10.1057/s41599-019-0256-3

[B48] Lyttelton T. Zang E. Musick K. (2020). *Gender Differences in Telecommuting and Implications for Inequality at Home and Work.* Rochester, NY: SSRN.

[B49] Machado L. S. Perlin M. Soletti R. C. e Silva L. K. R. Schwartz I. V. D. Seixas A. (2019). Parent in science: the impact of parenthood on the scientific career in Brazil. In *Proceedings of the 2nd International Workshop on Gender Equality in Software Engineering, GE ’19* (Montreal, QC: IEEE), 37–40.

[B50] Malisch J. L. Harris B. N. Sherrer S. M. Lewis K. A. Shepherd S. L. McCarthy P. C. (2020). Opinion: in the wake of COVID-19, academia needs new solutions to ensure gender equity. *Proc. Natl. Acad. Sci.U. S. A.* 117 15378–15381. 10.1073/pnas.2010636117 32554503 PMC7354923

[B51] Mason M. A. Goulden M. (2004). Marriage and baby blues: redefining gender equity in the academy. *Ann. Am. Acad. Polit. Soc. Sci.* 596 86–103. 10.1177/0002716204268744

[B52] McFarland J. Hussar B. Zhang J. Wang X. Wang K. Hein S. (2019). *The Condition Of Education 2019 (nces 2019-144). Tech. Rep.* Washington, DC: U.S. Department of Education.

[B53] McGee E. O. Bentley L. (2017). The troubled success of black women in stem. *Cogn. Instr.* 35 265–289.

[B54] MIT Committee on Women Faculty in the School of Science (1999). *A Study of the Status of Women Faculty in Science at MIT*, Vol. 9. Cambridge, MA: The MIT Faculty Newsletter

[B55] Morcelle V. Freitas G. Ludwig Z. M. D. C. (2019). From school to university: an overview on stem (science, technology, engineering and mathematics) gender in Brazil. *Quarks Braz. Electron. J. Phys. Chem. Mater. Sci.* 1 40–52. 10.34019/2674-9688.2019.v1.28228

[B56] Morgan F. (2015). The motherhood penalty and its impact of the career decisions of working women. *J. Marriage Fam.* 76 56–72. 10.13140/RG.2.1.3070.9288 PMC4041155 24904185

[B57] Moss-Racusin C. A. Dovidio J. F. Brescoll V. L. Graham M. J. Handelsman J. (2012). Science faculty’s subtle gender biases favor male students. *Proc. Natl. Acad. Sci.U. S. A.* 109 16474–16479. 10.1073/pnas.1211286109 22988126 PMC3478626

[B58] Myers K. R. Tham W.Y. Yin Y. Cohodes N. Thursby J.G. Thursby M.C. (2020). Unequal effects of the covid-19 pandemic on scientists. *Nat. Hum. Behav.* 4 880–883. 10.1038/s41562-020-0921-y 32669671

[B59] National Science Foundation (2015). *Science and Engineering Degrees, by Race/Ethnicity of Recipients: 2002–12. Detailed Statistical Tables NSF 15-321.* Alexandria, VA: National Science Foundation.

[B60] Nielsen M. W. Alegria S. Börjeson L. Etzkowitz H. Falk-Krzesinski H.J. Joshi A. (2017). Opinion: gender diversity leads to better science. *Proc. Natl. Acad. Sci.U. S. A.* 114 1740–1742. 10.1073/pnas.1700616114 28228604 PMC5338420

[B61] Noy C. (2008). Sampling knowledge: the hermeneutics of snowball sampling in qualitative research. *Int. J. Soc. Res. Methodol.* 11 327–344. 10.1080/13645570701401305

[B62] Ong M. Smith J. M. Ko L. T. (2018). Counterspaces for women of color in STEM higher education: marginal and central spaces for persistence and success. *J. Res. Sci. Teach.* 55 206–245. 10.1002/tea.21417

[B63] Peres I. T. Bastos L. S. L. Gelli J. G. M. Marchesi J. F. Dantas L. F. Antunes B. B. P. (2021). Sociodemographic factors associated with COVID-19 in-hospital mortality in Brazil. *Public Health* 192 15–20. 10.1016/j.puhe.2021.01.005 33607516 PMC7836512

[B64] Pohlhaus J. R. Jiang H. Wagner R. M. Schaffer W. T. Pinn V. W. (2011). Sex differences in application, success, and funding rates for NIH extramural programs. *Acad. Med.* 86 759–767. 10.1097/acm.0b013e31821836ff 21512358 PMC3379556

[B65] Power K. (2020). The covid-19 pandemic has increased the care burden of women and families. *Sustain. Sci. Pract. Policy* 16 67–73. 10.1080/15487733.2020.1776561

[B66] Reuben E. Sapienza P. Zingales L. (2014). How stereotypes impair women’s careers in science. *Proc. Natl. Acad. Sci.U. S. A.* 111 4403–4408. 10.1073/pnas.1314788111 24616490 PMC3970474

[B67] Rhoads S. E. Rhoads C. H. (2012). Gender roles and infant/toddler care: male and female professors on the tenure track. *J. Soc. Evol. Cult. Psychol.* 6 13–31. 10.1037/h0099227

[B68] Roberts L. M. McCluney C. L. (2020). *Working from Home While Black.* Boston, MA: Harvard Business Review Home.

[B69] Sallee M. Ward K. Wolf-Wendel L. (2016). Can anyone have it all? gendered views on parenting and academic careers. *Innov. High. Educ.* 41 187–202. 10.1007/s10755-015-9345-4

[B70] Shen H. (2013). Inequality quantified: mind the gender gap. *Nature* 495 22–24. 10.1038/495022a 23467149

[B71] Smith G. (2008). *Does Gender Influence Online Survey Participation: A Record-Linkage Analysis of University Faculty Online Survey Response Behavior. Tech. Rep.* San Jose, CA: San Jose State University.

[B72] Smith W. A. Allen W. R. Danley L. L. (2007). “Assume the position … you fit the description”. *Am. Behav. Sci.* 51 551–578. 10.1177/0002764207307742

[B73] Staniscuaski F. Reichert F. Werneck F. P. de Oliveira L. Mello-Carpes P. B Soletti R. C. (2020). Impact of covid-19 on academic mothers. *Science* 368 724–724. 10.1126/science.abc2740 32409466

[B74] Sullivan C. Lewis S. (2001). Home-based telework, gender, and the synchronization of work and family: perspectives of teleworkers and their co-residents. *Gend. Work Organ.* 8 123–145. 10.1111/1468-0432.00125

[B75] Treviño L. J. Gomez-Mejia L. R. Balkin D. B. Franklin G. Mixon J. (2018). Meritocracies or masculinities? the differential allocation of named professorships by gender in the academy. *J. Manag.* 44 972–1000. 10.1177/0149206315599216

[B76] Trower C. A. Chait R. P. (2002). Faculty diversity, too little for too long. *Harv. Mag.* 104 33–38.

[B77] Valentova J. V. Otta E. Silva M. L. McElligott A. G. (2017). Underrepresentation of women in the senior levels of brazilian science. *PeerJ* 5:e4000. 10.7717/peerj.4000 29302384 PMC5741063

[B78] van den Brink M. Benschop Y. (2012). Gender practices in the construction of academic excellence: sheep with five legs. *Organization* 19 507–524. 10.1177/1350508411414293

[B79] Verniers C. Vala J. (2018). Justifying gender discrimination in the workplace: the mediating role of motherhood myths. *PLoS One* 13 1–23. 10.1371/journal.pone.0190657 29315326 PMC5760038

[B80] Viglione G. (2020). Are women publishing less during the pandemic? here’s what the data say. *Nature* 581 365–366. 10.1038/d41586-020-01294-9 32433639

[B81] Vincent-Lamarre P. Sugimoto C. R. Larivière V. (2020). *The Decline of Women’s Research Production During the Coronavirus Pandemic.* Zeuthen: Nature Index

[B82] Wenham C. Smith J. Morgan R. Gender and COVID-19 Working Group (2020). Covid-19: the gendered impacts of the outbreak. *Lancet* 395 846—848. 10.1016/S0140-6736(20)30526-2 32151325 PMC7124625

[B83] Wenneras C. Wold A. (1997). Nepotism and sexism in peer-review. *Nature* 387 341–343. 10.1038/387341a0 9163412

[B84] Whittington K. B. (2011). Mothers of invention: gender, motherhood, and new dimensions of productivity in the science profession. *Work. Occup.* 38 417–456. 10.1177/0730888411414529

[B85] Williams W. M. Ceci S. J. (2012). When scientists choose motherhood: a single factor goes a long way in explaining the dearth of women in math-intensive fields. how can we address it? *Am. Sci.* 100 138–145. 10.1511/2012.95.138 24596430 PMC3939045

[B86] World Health Organization (2020). *Overview of Public Health and Social Measures in the Context of Covid-19: Interim Guidance, 18 May 2020. Technical Documents.* Geneva: World Health Organization.

